# Dysphoric Mood States are Related to Sensitivity to Temporal Changes in Contingency

**DOI:** 10.3389/fpsyg.2012.00368

**Published:** 2012-09-27

**Authors:** Rachel M. Msetfi, Robin A. Murphy, Diana E. Kornbrot

**Affiliations:** ^1^Department of Psychology, University of LimerickLimerick, Ireland; ^2^Department of Experimental Psychology, University of OxfordOxford, UK; ^3^School of Psychology, University of HertfordshireHatfield, UK

**Keywords:** causality, contingency, reinforcement, matching, maximization, learning, depression, depressive realism

## Abstract

A controversial finding in the field of causal learning is that mood contributes to the accuracy of perceptions of uncorrelated relationships. When asked to report the degree of control between an action and its outcome, people with dysphoria or depression are claimed to be more realistic in reporting non-contingency (e.g., Alloy and Abramson, 1979). The strongest evidence for this depressive realism (DR) effect is derived from data collected with experimental procedures in which the dependent variables are verbal or written ratings of contingency or cause, and, perhaps more importantly, the independent variable in these procedures may be ambiguous and difficult to define. In order to address these possible confounds, we used a two-response free-operant causal learning task in which the dependent measures were performance based. Participants were required to respond to maximize the occurrence of a temporally contiguous outcome that was programmed with different probabilities, which also varied temporally across two responses. Dysphoric participants were more sensitive to the changing outcome contingencies than controls even though they responded at a similar rate. During probe trials, in which the outcome was masked, their performance recovered more quickly than that of the control group. These data provide unexpected support for the DR hypothesis suggesting that dysphoria is associated with heightened sensitivity to temporal shifts in contingency.

## Introduction

Our perception of the effectiveness of our actions to elicit their consequences shapes our sense of volition (Neuringer and Jensen, 2010) and personal agency (Bandura, 1982) and may be related to mental health. People who are depressed often possess symptoms that resemble personal helplessness, yet it has been claimed, perhaps paradoxically, that they may also be more sensitive to the causal consequences of their behavior than others (Alloy and Abramson, 1979; Martin et al., 1984; Alloy et al., 1985; Benassi and Mahler, 1985; Vasquez, 1987). The strongest evidence for this depressive realism (DR) effect, involves data from the contingency judgment task (Dobson and Franche, 1989). This task requires the participant to make an arbitrary response and make a verbal or written judgment of the statistical relation between the response and an arbitrary outcome. However, there are possible problems with this method as an objective measure of people’s understanding of causal relationships. Here, we test mood related differences in causal learning using a less subjective measure of causal learning, a behavioral test of contingency and contiguity sensitivity measured over time, a method often used to study contingency sensitivity (e.g., Thomas, 1981; Dickinson et al., 1992) and timing sensitivity (Chiang et al., 2000) in animals. First, we briefly review DR studies before describing how the present experiment will address interpretative issues with extant measures.

The term DR originates from a series of studies carried out by Alloy and Abramson (1979). Student participants were given opportunities to press or not to press a button and observe whether an outcome (light) was temporally contingent upon their actions (key press). The programmed contingency between the action and the outcome can be formally described by the Δ*P* measure (Allan, 1980). Δ*P* expresses the strength of the relationship in terms of a number between −1 and +1, allowing for negative relationships. It is calculated as the difference between the conditional probabilities of the outcome following an action [*p*(light|press)] and following no action [*p*(light|no press)]. In most experiments, participants’ numeric judgments of their control over the outcome are then analyzed for consistency with the programed contingency. Indeed, much research has been conducted to determine the extent to which Δ*P*, as a formal model of contingency learning, is an accurate predictor of judgments (e.g., Jenkins and Ward, 1965; Allan and Jenkins, 1980; Chatlosh et al., 1985; Wasserman et al., 1993).

However, Alloy and Abramson’s (1979) aim was not so much to test the model but to assess the relative accuracy of judgments made by student participants, who were either categorized as mildly depressed or not depressed. A range of conditions and manipulations were tested across a series of experiments, though it was two critical conditions that engendered differences between the two mood groups. These were conditions where the frequency of outcomes was varied (25 versus 75% of trials included outcomes) but the difference between the two conditional probabilities and degree of control was always zero (Δ*P* = 0). Judgments made by the depressed participants reflected this contingency and in both conditions were close to zero, suggesting that they recognized their lack of control. Judgments made by the non-depressed participants, although low in the 25% condition, were higher in the 75% condition and were consistent with the perception of a moderate degree of control.

Based on these findings (Alloy and Abramson, 1979), and subsequent replications of the effect (Martin et al., 1984; Alloy et al., 1985; Benassi and Mahler, 1985; Vasquez, 1987), the general conclusions were that depressed people were realistic about control whereas the non-depressed were optimistic in their perceptions of causal efficacy. This evidence is considered to be strong largely because Δ*P* is regarded as an accurate objective measure of control against which to assess people’s ratings (Dobson and Franche, 1989; Ackermann and DeRubeis, 1991; Haaga and Beck, 1995).

However, the interpretation of such findings as indicating realism is based on the assumption that the experimentally programmed Δ*P* (Δ*P*_Prog_) and the Δ*P* experienced by the participant (Experienced DP: Δ*P*_Exp_) are the same or at least very similar. This may not be the case (Msetfi et al., 2005, 2007; Murphy et al., 2005). For example, Δ*P*_Prog_ is defined as the difference between two conditional probabilities. The first, *p*(Outcome|Action), is clear as it is defined by the participants’ responses. However, the second probability, *p*(Outcome|no Action), is ambiguous to manipulate experimentally because it is determined by the frequency of not responding. It is certainly not clear how non-events are defined over time and therefore their psychological frequency, as opposed to that which the experimenter programs and counts, is unknown (Msetfi et al., 2005, 2007). Thus the Δ*P*_Exp_ is also unknown in the case of contingencies where the *p*(Outcome|noAction) > 0. A stronger test of DR might involve conditions in which the experienced conditional probabilities and Δ*P*_Exp_ were under greater experimenter control.

Furthermore, other factors, such as response rate variability, can influence the Δ*P*_Exp_. Δ*P*_Exp_ is determined, to some extent, by the relative tendency to respond and to withhold responding. For instance, during 20 possible action opportunities if a participant responds 18 times and withholds responding 2 times, this sets limits on the range of possible contingencies that might be experienced. Some participants tend to respond more while others respond less even when instructed to sample both situations similarly. In extreme cases, the participant might experience only the *p*(Outcome|Action) or the *p*(Outcome|noAction) rather than both as intended by the experimenter (Matute, 1996) and a skewed Δ*P*_Exp_ depending on the programming method used (Matute, 1996; see also Hannah and Beneteau, 2009). This is a crucial issue for any experiment designed to test sensitivity to actual relationships. In fact, Matute (1996) has argued that the DR effect might occur simply because the depressed respond less than the non-depressed who respond at high rates and experience a more positive contingency. This interpretation suggests that the DR effect is a result of response rate differences changing the Δ*P*_Exp_.

Another obstacle to assessing perceptions of contingency involves the dependent variable in these studies. Many studies rely on participants’ explicit verbal or written judgments about their perception of control over outcomes using Likert or similar numerical scales. An alternative method we explore here is a performance measure (see also Hannah and Beneteau, 2009). There is some reason to suspect that the two measures might not elicit the same judgment. Verbal judgments may be more sensitive to disruption (Allan et al., 2005) and representative of people’s willingness to predict that an outcome will occur rather than their perception of the overall contingency (Allan et al., 2007).

In summary, we have identified three aspects of the standard contingency learning procedure used in DR studies, that may lead to equivocal or possibly misleading data with relation to the DR hypothesis. These are (i) the ambiguity of Δ*P*_Prog_ where *p*(Outcome|no Action) > 0; (ii) response rate variability which may affect Δ*P*; and (iii) reliance on explicit judgments of control as dependent measures which are assumed to reflect contingency sensitivity.

The current study was designed to address these issues. The primary change involved examining not the accuracy of judgments of contingency but how effective participants’ responses were in causing an outcome to occur. The procedure is based on a standard instrumental or free-operant procedure in which participants are instructed to cause an outcome to occur (e.g., flash of light on a computer screen) as many times as possible. Note that in this procedure the outcome never occurs in the absence of the response and, no matter what the response rate, the *p*(light|no press) is always zero. Also rather than assess contingency as the difference in perceived effectiveness of responding or not responding, the procedure required participants to learn which of two responses was more effective and that this would vary over the course of the trial. This means that responding should be maintained on a given action while it is more contingent, but that it should shift between responses as the contingencies change.

However, in the two-response procedure we used in the present experiment, there are two possible behavioral strategies that people might employ. Previous research indicates that under similar conditions, people tend to “match” their responses to the outcome contingencies (e.g., Chatlosh et al., 1985; Koehler and James, 2009). This is consistent with Herrnstein’s (1961) Matching Law in which the relative probability of responding on each of the two behavioral choices matches the probability of reinforcement. Alternatively participants might employ an all or nothing maximization strategy which might actually be more effective in producing outcomes. In other words, when *p*(flash|response_1_) > *p*(flash|response_2_), then the most effective means of producing the maximum number of light flashes is to make only response_1_ and not response_2_. In the conditions tested here, the maximization strategy could thus be thought of as the most effective and normative option in comparison to contingency matching strategies.

In addition, behavior that tracks changes in the probability of the light flash might be claimed to reflect sensitivity to correlated and uncorrelated actions as well as adjustment speed. Successful performance on the task involves being sensitive to which of the two actions is more contingent with the outcome and then changing behavior to reflect the change in contingency over time. Indeed, sensitivity to shifts in the temporal predictiveness of actions for individual cues are argued to be an important cue to causality (Greville and Buehner, 2010). It spite of this, studies exploring the effects of depression on causal learning have not so far included time based responses to contingency. Given, however, that mild depression has been linked to a slowing down in the perception of time itself (e.g., Gill and Droit Volet, 2009), possibly through attentional mechanisms (Msetfi et al., 2012), participants with depression may be less sensitive to temporal shifts in reinforcement.

This leaves us with some interesting and testable predictions. Across a number of conditions, mildly depressed people are more sensitive to uncorrelated contingencies than controls (e.g., Alloy and Abramson, 1979). However, there is little evidence to suggest that sensitivity leads to more effective behavior. For example, related research on learned helpless suggests that depression is associated with passive behavior in the face of positive contingencies (e.g., Hiroto, 1974; Maier and Seligman, 1976). Therefore, we might hypothesize that, if realistic, mildly depressed participants’ response rate probabilities will be more similar to the programmed contingencies than those of the control group. However, we would also hypothesize that the response rates of controls will be greater and produce a greater frequency of light flashes. Based on Matute and her colleagues’ work on the link between response rates and DR (Matute, 1996; Blanco et al., 2009), controls will respond at higher rates or adopt a maximization strategy, and, consequently, experience more light flashes than the dysphoric group. Finally, based on research into the slowing effects of depression on time perception, we hypothesize that depressed participants will respond later to the switch in contingencies than the control group (e.g., Tysk, 1984; Bschor et al., 2004; Gill and Droit Volet, 2009; Msetfi et al., 2012).

## Materials and Methods

### Participants

University students completed the Beck Depression Inventory (BDI; Beck et al., 1961) before being invited to participate and again during participation. All participants gave consent after being informed as the nature of the study. The final sample comprised forty-eight participants who were assigned to the dysphoric (*n* = 24) or control groups (*n* = 24) on the basis of their BDI scores. As with the majority of DR research (e.g., Alloy and Abramson, 1979; Msetfi et al., 2005), scores of 9 or above indicated dysphoric mood and scores of 8 or below indicated no depression and membership of the control group. The groups were matched on demographic variables, gender, handedness, age, years of education, pre-morbid IQ measured by the National Adult Reading Test (NART; Nelson, 1982), and short term memory capacity (Digit span; Lezak, 1995). All participants were right handed and all between group *t*-tests carried out on demographic were not were not reliable (all *t* < 1.14). However, as expected, the dysphoric group had significantly higher BDI scores (*M* = 15.6, SE = 1.6) than the control group (*M* = 4.4, SE = 0.5: *t*(46) = 6.56, *p* < 0.001).

### Procedure

Participants were briefed about the nature of the experiment and read an information sheet. Participants completed the digit span test, the NART, and the BDI. Instructions for the experimental task were then presented on a computer screen, which participants were able to read self-paced. The full instructions are shown in the Appendix. In the instructions, participants were asked to maximize the occurrence of a brief light flash on the computer screen by pressing two on-screen buttons as many times as they chose to during each experimental trial. The button on the left could be pressed using the left “tab” key and the button on the right using the “return” key on the computer keyboard. For half the participants, response_1_ was the left on the computer keyboard with response_2_ on the right. For the other half of the sample, this positioning was reversed. Buttons were not to be pressed simultaneously or held in the on position.

Each trial was 50 s long and separated by a 10 s inter-trial interval. During the first 25 s of each trial, 85% of presses on response_1_ – the “early” button – were reinforced immediately with a light flash, while 15% of presses on response_2_ – the “late” button were reinforced. Reinforcement sequences were generated randomly for each participant. In addition, the outcome contingencies switched buttons after 25 s in the middle of each trial. All light flashes lasted 100 ms with no delay between the button press and the flash. Dependent measures were response rates and the probability of pressing the late button during each 5 s time segment of every experimental trial [*p*(late) = *F*(late)/*F*(early) + *F*(late)].

There were a total of 18 trials in the procedure. However, participants were told that there would be some probe trials where the light would be hidden from them, but that they should use what they had already learned in order to make the light flash as many times as possible (trials: 9, 12, 15, and 18). An on-screen message at the end of each trial recorded the number of light flashes during that trial. Finally, participants were debriefed, and paid a nominal fee for their participation.

## Results

The probability of pressing the late button – *p*(late) – was calculated for every 5 s time segment for each participant, across reinforced learning trials and also across masked probe trials. The analyses of reinforced and probe trial data are reported separately below, and an alpha level of 0.05 was used in all analyses unless stated otherwise.

### Reinforced learning trials

The *p*(late) for each 5 s time segment was analyzed using a mixed (14 × 10) × 2 factorial analysis of variance with trial (1–14) and time segment (5–50 s) as within subjects variables and mood (dysphoric, control) as the between subjects variable. For brevity and simplicity, data are shown in Table 1 for each time segment averaged over experimental trials.

**Table 1:** **Mean probability of responding on the late button [*p*(late)] for the control and dysphoric groups during each 5 s time segment averaged over 14 reinforced trials**.

Time (s)	*M*	SE	Contingency comparison0.15 or 0.85		Maximization comparison0 or 1	
			*t*	*p*	*t*	*p*
**CONTROL GROUP**						
5	0.173	0.028	0.819	0.421	6.182	<0.001
10	0.135	0.027	−0.563	0.579	4.921	<0.001
15	0.126	0.027	−0.874	0.391	4.65	<0.001
20	0.112	0.027	−1.379	0.181	4.128	<0.001
25	0.119	0.026	−1.194	0.245	4.513	<0.001
30	0.574	0.019	−14.605	<0.001	−22.532	<0.001
35	0.861	0.027	0.42	0.678	−5.094	<0.001
40	0.879	0.027	1.062	0.299	−4.448	<0.001
45	0.89	0.025	1.579	0.128	−4.326	<0.001
50	0.896	0.023	1.964	0.062	−4.477	<0.001
**DYSPHORIC GROUP**						
5	0.211	0.040	1.538	0.138	5.291	<0.001
10	0.177	0.035	0.753	0.459	5.016	<0.001
15	0.154	0.036	0.125	0.902	4.463	<0.001
20	0.158	0.033	0.252	0.803	4.789	<0.001
25	0.171	0.035	0.587	0.563	4.779	<0.001
30	0.627	0.015	−15.246	<0.001	25.487	<0.001
35	0.874	0.026	0.942	0.356	−4.918	<0.001
40	0.882	0.027	1.178	0.251	−4.403	<0.001
45	0.884	0.025	1.404	0.174	−4.716	<0.001
50	0.868	0.025	0.716	0.481	−5.34	<0.001

*These comparisons were made using single sample *t*-tests and the alpha level was ameliorated to α = 0.00125 for 40 comparisons*.

Response probabilities did change across time segments, *F*(9, 414) = 324.51, *p* < 0.001, η^2^ = 0.88, MSE = 0.273. Although the effect of trial was not reliable, *F*(13, 598) = 1.495, *p* = 0.114, MSE = 0.048, the trial by time segment interaction was significant, *F*(117, 5382) = 6.93, *p* < 0.001, η^2^ = 0.13, MSE = 0.024, with response probabilities increasingly matching programmed contingencies as learning progressed. This trend did not depend on mood group however, as the three-way interaction between trial, time segment, and mood group was not significant (*F* < 1). However, there was a significant main effect of mood *F*(1, 46) = 4.43, *p* = 0.041, η^2^ = 0.088, MSE = 0.221. Dysphoric participants *p*(late) was higher (*M* = 0.501, SE = 0.01) than controls (*M* = 0.476, SE = 0.01) throughout the reinforced learning trials.

The main effect of mood on response probabilities does not indicate whether participants were using a contingency matching strategy or a maximization strategy. Consider that if responses are distributed across buttons in a manner consistent with the programmed contingency (0.15 and 0.85) or maximization (0 and 1), then the response probability should average out at 0.50 over the course of each experimental trial. As dysphoric participants responded on the late button with a probability of 0.500 and this was significantly higher than controls, who responded at a probability of 0.476, this is evidence of responding which is closer to one of those strategies. In order to explore this further, the *p*(late) was compared to the DP programmed at the same time points and to values consistent with a maximization strategy (see Table 1) using a series of single samples *t*-tests. The alpha level for these tests was ameliorated to account for multiple comparisons, where α = 0.05/40 comparisons = 0.00125.

Table 1 shows that for both groups, response probabilities during 9 of the 10 time segments were not significantly different from the programmed contingencies but were significantly different from maximization probabilities throughout. Thus participants’ responses matched contingencies rather than being consistent with the more effective maximization strategy. However, as the dysphoric group achieved an average response probability of 0.500 overall (see above), which was significantly higher than that of controls (*p* = 0.04), this is suggestive of a general increased probability sensitivity in the dysphoric group. Greater sensitivity could also be indicated by a more rapid switch in response probabilities between the two buttons at 25 s. However, as there were no significant interactions involving mood and time segment, *F* < 1, there was no evidence for any mood related changes in this type of contingency sensitivity.

In order to check whether the group effect on response probabilities reported above was related to mood related changes in response propensity, we also examined absolute response frequencies. The data were then analyzed using a similar analysis of variance strategy to that described above, with trial (14), time segment (10), and button (early, late) as within subjects variables and mood as the between subjects variable and are shown below in Table 2 averaged over trials and button. Overall response frequencies increased over trials, from an average of 140.50 (SE = 11.10) on Trial 1 to 279.5 (SE = 11.31) on Trial 14, *F*(13, 598) = 45.17, *p* < 0.001, MSE = 76.42, and over time segments, *F*(9, 414) = 21.20, *p* < 0.001, MSE = 20.29. The dysphoric group responded on average 257.1 times during each trial (SE = 15.1), while controls made fewer responses (*M* = 229.1, SE = 15.1). However, the mood effect was not reliable, *F*(1, 46) = 1.73, *p* = 0.195.

**Table 2:** **Absolute frequency of response for the control and dysphoric groups during each 5 s time segment averaged over the early and late buttons and the 14 reinforced trials**.

Time (s)	Control group		Dysphoric group	
	*M*	SE	*M*	SE
5	9.73	0.844	11.49	0.701
10	11.9	0.900	13.2	0.787
15	11.7	0.826	12.96	0.749
20	11.57	0.789	12.73	0.718
25	11.36	0.768	12.71	0.712
30	11.67	0.812	12.86	0.737
35	11.94	0.772	13.22	0.731
40	11.64	0.715	13.13	0.787
45	11.56	0.707	13.13	0.804
50	11.46	0.703	13.11	0.801

Finally, response frequency data was used to calculate a measure of the effectiveness of responding over reinforced trials in terms of light flashes produced [effectiveness = First 25 s (*F* Early × 0.85) + (*F* Late × 0.15); Second 25 s (*F* Early × 0.15) + (*F* Late × 0.85)]. These data were analyzed using a mixed analysis of variance, with trials (14) and trial half (first 25 s, second 25 s) as the repeated measures factors. Mood was the between subjects factor. Response effectiveness improved over trials, *F*(13, 32) = 19.35, *p* < 0.001. Although the dysphoric group produced more flashes (*M* = 93.72, SE = 5.1) than controls (*M* = 84.9, SE = 5.1), the mood effect was again not significant, *F*(1, 44) = 1.47, *p* = 0.231, nor were any of the interactions involving mood.

Response rate and response effectiveness data from reinforced trials therefore shows that controls did not produce more responses than the dysphoric group or receive more light flashes.

### Masked probe trials

As with reinforced trials, we calculated *p*(late) values for each time segment of the four masked probe trials. These data are shown in Table 3, along with the average *p*(late) across each probe trial. Inspection of these data shows that, in spite of the light flashes being masked, participants were able to maintain appropriate performance and switch responding from the early to the late buttons midway through the each trial.

**Table 3:** **Mean probability of responding on the late button [*p*(late)] for the control and dysphoric groups during each 5 s time segment and on average (labeled *M*) for each the four masked probe trials**.

Time segment in s	Probe 1		Probe 2		Probe 3		Probe 4	
	*M*	SE	*M*	SE	*M*	SE	*M*	SE
**CONTROL GROUP**								
5	0.028	0.022	0.079	0.044	0.071	0.041	0.032	0.020
10	0.144	0.039	0.164	0.052	0.134	0.052	0.098	0.048
15	0.134	0.041	0.26	0.07	0.142	0.052	0.135	0.049
20	0.201	0.051	0.381	0.082	0.179	0.059	0.214	0.068
25	0.322	0.078	0.519	0.084	0.343	0.079	0.332	0.058
30	0.505	0.081	0.702	0.066	0.642	0.072	0.75	0.068
35	0.668	0.056	0.774	0.066	0.809	0.07	0.886	0.047
40	0.724	0.064	0.727	0.069	0.905	0.044	0.892	0.055
45	0.719	0.063	0.726	0.066	0.921	0.037	0.914	0.046
50	0.74	0.062	0.708	0.076	0.828	0.07	0.873	0.053
***M***	0.419*	**0.023**	0.504	0.025	0.498	0.03	0.513	0.021
**DYSPHORIC GROUP**								
5	0.068	0.039	0.167	0.061	0.164	0.054	0.14	0.052
10	0.271	0.066	0.214	0.07	0.111	0.045	0.148	0.052
15	0.221	0.056	0.179	0.065	0.138	0.052	0.18	0.063
20	0.291	0.061	0.245	0.057	0.19	0.06	0.189	0.063
25	0.491	0.07	0.433	0.076	0.37	0.074	0.259	0.069
30	0.651	0.069	0.62	0.084	0.645	0.071	0.606	0.073
35	0.698	0.061	0.786	0.059	0.76	0.069	0.731	0.073
40	0.707	0.068	0.853	0.042	0.809	0.056	0.844	0.048
45	0.76	0.051	0.793	0.06	0.839	0.059	0.873	0.043
50	0.769	0.065	0.79	0.054	0.849	0.056	0.816	0.059
***M***	0.493*	**0.020**	0.508	0.021	0.488	0.026	0.479	0.022

Between group differences are emphasized in bold, *indicates the level of significance = 0.018.

The results of the mixed factorial analysis of variance were consistent with these observations, with the effects of trial, time segment and the trial by time segment interaction being significant, all *F* > 2.62, all *p* < 0.04. Of more interest, however, were mood effects. The trial by mood interaction was significant, *F*(3, 138) = 3.28, *p* = 0.023, η^2^ = 0.07, MSE = 0.08. Tests of the simple effects of mood on *p*(late) during each probe trial showed that the control group responded significantly less than the dysphoric group overall during the first probe trial, *p* = 0.018, but not the second third or fourth probe trials, all *p*s > 0.26.

**Figure 1:**
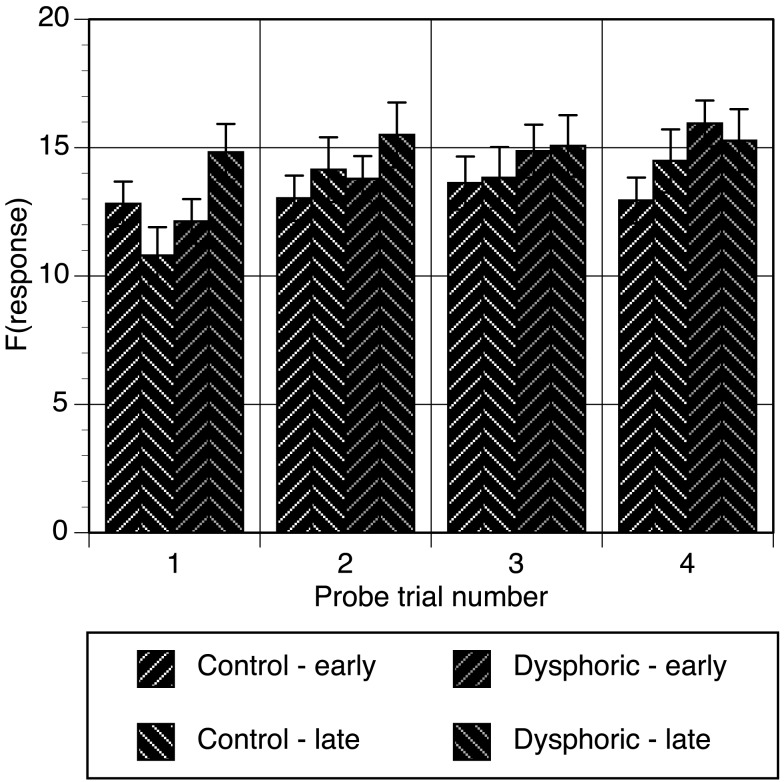
**Response frequency on the early and late buttons during each probe trial averaged over time segment for the control and dysphoric groups**. Error bars correspond to the standard error of the mean.

Response frequencies on each button were also examined for each time segment of each probe trial and are shown in Figure 1 averaged over time segment. The data were analyzed with trial (4), time segment (10), and button (early, late) as within subjects variables and mood as the between subjects variable. As expected, response frequencies increased over the four probe trials, and also depended on button, and time segment, all *F*s > 5.31, all *p*s < 0.001. Of interest here were the effects involving mood. Figure 1 suggests that mood effects were present in the first probe trial but not subsequent trials. This observation was consistent with the significant mood by trial by button interaction, *F*(3, 138) = 4.29, *p* = 0.006, η^2^ = 0.085, MSE = 116.61. Further analysis of this interaction showed that the mood by button interaction was only significant in the first probe trial, *F*(1, 46) = 7.42, *p* = 0.009, η^2^ = 0.139, MSE = 178.92, and not the subsequent probe trials, all *F*s < 1.11, all *p*s > 0.29. The source of the mood difference in Probe trial 1 was revealed through simple effects analyses, and showed that the dysphoric group responded significantly higher on the late button than the early button, *p* = 0.03, and that this was at a significantly higher level than controls, *p* = 0.013.

**Figure 2:**
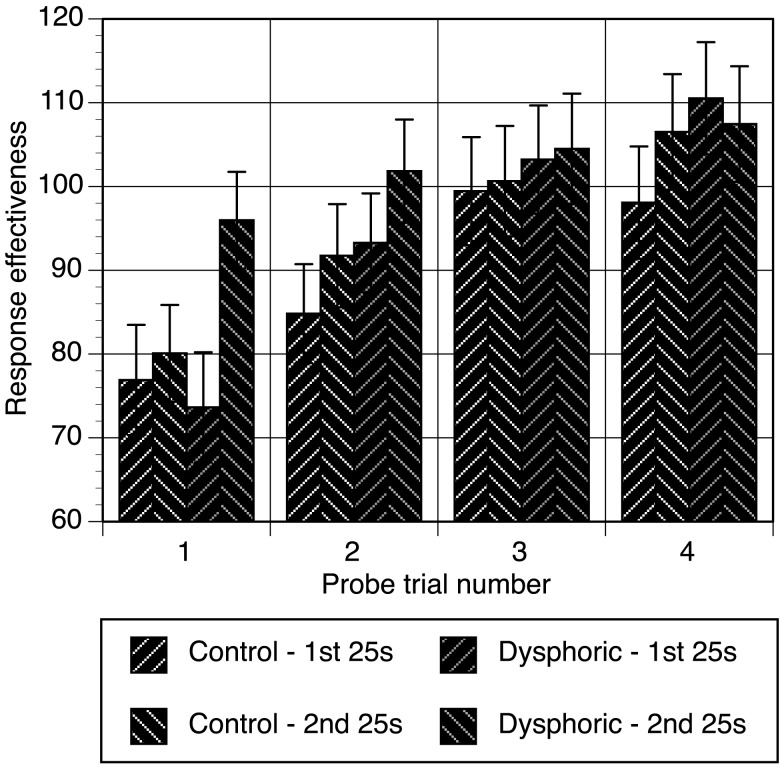
**Response effectiveness scores during the first 25 s and second 25 s of each probe trial for the control and dysphoric groups**. Error bars correspond to the standard error of the mean.

Response effectiveness scores were calculated for each probe trial (see Figure 2). These data were analyzed with a mixed analysis of variance with time segment (first 25 s, second 25 s), and trial number (1–4) as repeated measures factors. Mood was the between subjects factor. Response effectiveness increased significantly over the four probe trials, *F*(3, 138) = 29.49, *p* < 0.001, η^2^ = 0.39, MSE = 372.03, but depended on whether responses were made in the first or second half of the trial, *F*(3, 138) = 4.22, *p* = 0.007, η^2^ = 0.084, MSE = 156.24, and mood group, *F*(3, 138) = 6.15, *p* = 0.001, η^2^ = 0.118, MSE = 156.24. Further analysis of this three-way interaction between trial, time segment, and mood involved examining the simple interactions between time segment and mood group for each probe trial. The time by mood interaction was only significant for the first probe trial, *F*(1, 46) = 6.82, *p* = 0.012, η^2^ = 0.129, MSE = 322.79, but not subsequent probe trials, all *p*s > 0.18. Thus, probe trial 1 was the source of mood effects on response effectiveness, where there was no difference between mood groups in the first 25 s of the first probe trial, *p* = 0.73. However, the dysphoric group significantly improved their effectiveness by the second 25 s of the first probe trial, *p* < 0.001, and the difference between their effectiveness and that of the control group approached the level of significance, *p* = 0.058. The control group’s performance did not improve during the first probe trial, *p* = 0.54. This data indicates that the dysphoric group recovered from the detrimental effect on performance of masked trials more rapidly than controls.

The data from masked probe trials shows that the effects of mood were only evident on the first probe trial. The probability that the control group pressed the late button during this trial was lower in comparison to the dysphoric group. This between groups difference was due to the dysphoric group returning to higher levels of response frequency and response effectiveness in the second half of the first trial with their responses on the late button.

## Discussion

Participants in this study “matched” their responding to the programmed reinforcement contingencies (e.g., Chatlosh et al., 1985). They did not use what would have been a more effective, but more effortful, all or nothing maximization strategy (Koehler and James, 2009). In other words, they would have produced more outcomes had they made all responses on one button in the first half of each trial [*p*(early) = 1, *p*(late) = 0] and then switched to the other button during the second half [*p*(early) = 0, *p*(late) = 1]. In general, on reinforced trials, the dysphoric group matched their behavior more closely to the contingencies than controls. Although there was no evidence that the mood groups differed in the extent to which they responded to the temporal shift in reinforcement. When reinforcement was masked during the probe trials, both groups performance suffered initially, but the dysphoric group recovered more quickly. These findings will be discussed in more detail below in relation to the DR hypothesis and the methodological issues raised in the introduction.

In the introduction, we noted several possible methodological issues with DR research, carried out by ourselves and others (e.g., Alloy and Abramson, 1979; Msetfi et al., 2005), that has utilized standard contingency judgment procedures. These included the ambiguity of the Δ*P*_Prog_, especially in conditions with a non-zero *p*(light|no press) or long periods of inter-trial interval (Msetfi et al., 2005), and of the Δ*P*_Exp_, as a result of response rate fluctuations (e.g., Matute, 1996). Finally, explicit verbal or written judgments of control may produce a biased test of contingency sensitivity and the DR hypothesis (Allan et al., 2007).

We found that when participants were exposed to the reinforcement of the light flash during learning trials, the dysphoric group responded overall in a manner that was more consistent with the programmed contingencies than the controls. Consider that the programmed contingencies on each lever were Δ*P* = 0.85 and Δ*P* = 0.15, the average of which is 0.50 over the course of each experimental trial. Both groups behavior “matched” the programmed contingencies, but the dysphoric group produced an average *p*(late) of 0.5, consistent with the programmed average, and significantly higher than controls. In order to produce this pattern of probabilities, participants were required, not only to be sensitive to the programmed contingencies, but also to match their behavior rapidly and closely to the switch between contingencies midway through the trial. Although there is some evidence that people with depression are less sensitive to time passage (Msetfi et al., 2012), and perceive time to be passing more slowly (e.g., Gill and Droit Volet, 2009), there was no evidence here that dysphoria was associated any temporal insensitivity to reinforcement contingencies. In fact, to the contrary, it could be argued that improved matching is evidence of improved contingency sensitivity and perhaps DR in the dysphoric group.

This evidence for DR could however have been based on fluctuations in response frequency rather than any particular propensity to realism. It has been argued previously that response rate variability could produce deviations in the Δ*P*_Exp_, as well as the effectiveness of behavior. Indeed, helplessness and behavioral passivity is one of the classic symptoms of depressed mood. In this study, it was not possible for fluctuations in levels of behavior to influence the Δ*P*_Exp_, but it was possible that controls would respond at higher levels, and that their responses would therefore be more effective in producing outcomes, even if they were less realistic than the dysphoric group. However, we found no evidence that controls either responded at higher levels or received higher levels of reinforcement (Blanco et al., 2009). In fact, overall, the control group responded at a lower level than the dysphoric group. On reinforced trials, this difference was too subtle to produce a significant effect on response effectiveness. Thus, whereas the dysphoric group was more successful at contingency matching than controls, there were no between group differences on reinforced trials in how effective behavior was in producing as many light flashes as possible.

It was also important to examine responding in the absence of direct exposure to reinforcement. On masked probe trials, both groups responded appropriately and effectively, though performance did suffer initially from the absence of reinforcement. As hypothesized, between group differences were amplified in the absence of reinforcement. We had expected that realism, or an improved awareness of the causal effectiveness of actions, would result in less of a decrement in performance when reinforcement was withdrawn. However, although response effectiveness dropped for both groups in early probe trials, the speed of recovery from it depended on mood. For the control group, response effectiveness steadily improved over the four probe trials. The dysphoric group recovered rapidly by the second half of the first probe trial to formerly high levels. Essentially, the dysphoric group required less time to recover from the withdrawal of direct reinforcement. These results have several theoretical implications and suggest avenues that require further exploration.

For example, these data provide no support for the idea that depression is consistent with low response levels and reduced exposure to reinforcement (e.g., Lewinsohn and Libet, 1972; Lewinsohn and Graf, 1973). In fact, both dysphoric and control groups responded at equally high levels. This finding is also inconsistent with Blanco et al. (2009), who found that dysphoric participants, exposed to a zero contingency procedure with a high frequency of outcomes, responded less and made lower contingency ratings than controls. In the present experiment, participants were exposed to positive outcome probabilities in comparison to the zero contingencies Blanco et al. (2009) tested. Thus it might be that zero contingency conditions are a special case that produce lower response rates in dysphoric groups, which might itself be a particular form of the DR effect.

It is also interesting to note that, in the present study, dysphoric participants were less affected than controls by the withdrawal of reinforcement in probe trials. Although response effectiveness was reduced just like with controls, the effect did not last so long in the dysphoric group. The effectiveness of their behavior improved significantly by the second half of the first probe trial. One possible reason for this might be because people with dysphoria are less responsive or sensitive to reinforcement in the first place and less affected by its absence. This suggestion is consistent with negative relationships, reported in the normal population, between mood and reinforcer sensitivity (Glautier et al., 1998), antidepressant administration and increased sensitivity to outcomes (Murphy et al., 2012), and theoretical reinforcement sensitivity approaches to psychopathology (e.g., Gray, 1982).

Thus far, we have interpreted the current findings as evidence for DR. It should be noted however that, rather than evidence of good learning, probability matching as observed in the general population has been characterized as a non-normative tendency (West and Stanovich, 2003). In comparison to a considered and effective maximization strategy, contingency matching could be seen as a “mistake” based on a rapid response to the situation (Koehler and James, 2009). In fact, we hypothesized that controls might actually adopt a maximization strategy based on higher response rates. This was not the case. In this study, controls “matched” their responses to the programmed contingencies but not quite as consistently as the dysphoric group. However, it could be argued that the current results are not suggestive of improved learning in dysphoria but perhaps a stronger tendency toward less than normative responses.

In summary, we have found dysphoria to be associated with improved response-outcome contingency sensitivity using a time based contingency procedure. Participants in the dysphoric group produced responses that were more consistent with the programmed contingency over time than participants in the control group. The effectiveness of their responses also recovered more rapidly from the withdrawal of reinforcement. There was no evidence for a link between dysphoria and reduced response levels, experienced reinforcement contingencies or reduced sensitivity to temporally marked changes in contingency. The findings from this behavioral task are novel, as effective performance must involve sensitivity to contingencies that change over time and depression effects on causal learning have not previously been studied in this manner. These findings provide support for the DR hypothesis, though realism may not necessarily be indicative of normative behavior.

## Conflict of Interest Statement

The authors declare that the research was conducted in the absence of any commercial or financial relationships that could be construed as a potential conflict of interest.

## Appendix

### Instructions

The task instructions appeared on the computer screen, and participants were able to self pace through the instructions using the “carry on” button.

#### Screen 1

In this game you will see a picture of a light bulb appear on the screen beside two different buttons.

It will be your job to try to make the light switch on as many times as possible. You will be able to press the buttons next to the light bulb as many times as you wish in order to try to make the light come on.

#### Screen 2

When the light does switch on, it will appear as a brief flash and will switch off again immediately. We want this to happen as many times as possible.

There are several rules to this game however!

*You must not press both buttons at once.*

*You must not press and hold the buttons down.*

#### Screen 3

When the game starts, you will see the light bulb first of all.

Then after waiting for a few seconds, the two buttons will appear.

When you can see them, you can press them!

You can press the button on the left using “the tab key.”

You can press the button on the right using the return key.

#### Screen 4

There is no limit to how many times you can press the buttons (as long as you follow the rules).

Just keep in mind that your job is to make the light switch on as MANY times as possible.

#### Screen 5

When your first go at pressing the buttons is finished, the buttons will disappear from the screen.

There will be a short delay, after which the buttons will reappear and you can have another go. Each go at pressing the buttons is called a trial.

You will have quite a lot of trials in this button pressing game, and during each trial, you must follow the same rules.

#### Screen 6

During each trial, you will learn a lot about how to make the light flash. We will need to check what you have learned.

Therefore on some trials a message will appear on the screen saying “This is a test trial.” On a test trial, you will not see the light bulb on the screen. Even though you cannot see the light, you must carry on and press the buttons exactly as if the light bulb were on the screen.

#### Screen 7

Even though you will not be able to see the light on test trials, the experimenter will monitor how often the light flashes in order to collect data. Your aim on test trials is to use what you have learned to make the light flash as many times as possible.

There will only be a few test trials in the whole game, which will take approximately 15 min. When the game is finished, a message will appear on the screen. If you have any questions, please ask the experimenter now or say that you are ready to start the game.
